# An Account of the Diseases On-Board His Majesty's Ship Belliqueux, Capt. Byng, of 64 Guns and 491 Men, during a Voyage from China to England, between the 10th of February and the 8th of August, 1811

**Published:** 1812-10

**Authors:** R. W. Bampfield

**Affiliations:** H. M. S. Warrior, off Flushing


### For the Medical and Physical Journal.

*An* ACCOUNT *of the* DISEASES *on-board his* MAJESTY'S SHIP BELLIQUEUX, CAPT. BYNG, *of* 64 GUNS *and* 491 MEN, *during a* VOYAGE *from* CHINA *to* ENGLAND, *between the* 10*th of* FEBRUARY *and the* 8*th of* AUGUST, 1811.

| DISEASES. | Total put on the Sick List. | Total cured on the Passage. | Died. | Sent to a Naval Hospital. | On the List at the Period of Arrival. | Cured on-board in England. | Total cured on-board. |
|---|---|---|---|---|---|---|---|
| Febres Intermittentes | 2 | 1 | — | — | 1 | 1 | 2 |
| ——— Continuæ - - | 15 | 15 | — | — | — | — | 15 |
| Phlogosis - - - - | 8 | 8 | — | — | — | — | 8 |
| Abscessus - - - - | 14 | 14 | — | — | — | — | 14 |
| Ophthalmia - - - - | 2 | 2 | — | — | — | — | 2 |
| Cynanche Tonsill. - | 4 | 4 | — | — | — | — | 4 |
| ——— Parotidæa - | 2 | 2 | — | — | — | — | 2 |
| ——— Maligna - | 3 | 3 | — | — | — | — | 3 |
| Otalgia - - - - - | 2 | 2 | — | — | — | — | 2 |
| Hepatitis Acuta - - | 1 | 1 | — | — | — | — | 1 |
| ——— Chronica - | 7 | 5 | — | — | 2 | 2 | 7 |
| Dysenteria - - - - | 20 | 18 | — | — | 2 | 2 | 20 |
| Rheumatismus - - | 10 | 10 | — | — | — | — | 10 |
| Arthrodynia - - - | 2 | 2 | — | — | — | — | 2 |
| Lumbago - - - - | 3 | 3 | — | — | — | — | 3 |
| Hernia Humoralis - - | 2 | 2 | — | — | — | — | 2 |
| Pleurodyne - - - - | 1 | 1 | — | — | — | — | 1 |
| Hæmorrhagia Intestin. | 1 | 1 | — | — | — | — | 1 |
| Catarrhus - - - - | 7 | 7 | — | — | — | — | 7 |
| Bilis Secretio Acuta - | 24 | 24 | — | — | — | — | 24 |
| Dyspepsia - - - - | 3 | 3 | — | — | — | — | 3 |
| Epilepsia - - - - | 1 | 1 | — | — | — | — | 1 |
| Palpitatio - - - - | 1 | 1 | — | — | — | — | 1 |
| Colica - - - - - | 1 | 1 | — | — | — | — | 1 |
| Diarrhœa - - - - | 17 | 17 | — | — | — | — | 17 |
| Vermes - - - - - | 1 | 1 | — | — | — | — | 1 |
| Atrophia Cacochymica | 1 | — | — | 1 | — | — | — |
| ——— Scorbutica - | 1 | 1 | — | — | — | — | 1 |
| Syphilis - - - - - | 1 | — | — | 1 | — | — | — |
| *Scorbutus - - - - | 38 | 31 | — | 1 | 6 | 6 | 37 |
| Hemeralopia, (vulgo Ncytalopia) - - | 6 | 6 | — | — | — | — | 6 |
| Strictura Urethræ - - | 3 | 2 | — | — | 1 | — | 2 |
| Hernia - - - - - | 1 | Truss app. | — | — | — | — | — |
| Contusio - - - - | 12 | 12 | — | — | — | — | 12 |
| Luxatio Humeri - - | 2 | 2 | — | — | — | — | 2 |
| ——— Digitorum - | 1 | 1 | — | — | — | — | 1 |
| ——— Pollicis Pedis - | 1 | 1 | — | — | — | — | 1 |
| Vulnera - - - - - | 31 | 30 | — | — | 1 | 1 | 31 |
| Fractura Femoris - - | 1 | 1 | — | — | — | — | 1 |
| ——VertebræCervical. | 1 | 1 | 1 | — | — | — | — |
| ——Maxillæ Inferioris | 1 | 1 | — | — | — | — | 1 |
| Ambustio Radiis Solis | 10 | 10 | — | — | — | — | 10 |
| Total - - | 265 | 248 | 1 | 3 | 13 | 12 | 259 |

* Besides the number of scorbutics placed on the sick lists, and exempted from duty, about 200 slighter cases were treated, who were recommended to continue their usual exercise, enjoined in the performance of their duty on-board.

His

His Majesty's ship Belliqueux had been in the East Indies five years; and, previous to leaving India for China, in June, 1810, fifty of her healthy men had been exchanged for a similar number of seamen from other ships, whose constitutions had been much impaired by a residence of ten years and upwards in the oriental tropics, and there were ordered on-board 32 men, invalided from various diseases. The Belliqueux arrived in China in September 1810, and remained at Chicenpee, lat. 22° 44′ N. lon. 115° E. near the Boca Tigris, until the 10th of February, 1811. A degree of cold prevailed during part of the month of November, and the months of December, January, and February, which we had been utter strangers to in India, and which was suddenly introduced by the north-east monsoon. The thermometer in the shade ranged from 40 to 62 degrees of Fahrenheit in the day-time, and at night sometimes sunk to the freezing point. The ship's company obtained more nutritive and finer beef than they were accustomed to meet in India, and the improved state of horticulture in China furnished ample supplies of fine vegetables and fruit, which, with the advantages of a cold climate, improved the general health and appearance of all the men, notwithstanding the sudden vicissitude from heat to cold occasioned several inflammatory complaints.

The experience that had been acquired by our long voyages in tropical regions, had fully proved that scurvy, contrary to received opinion,* was much sooner induced at sea in a torrid than in a temperate or frigid zone, and was not only highly fatal itself, but impeded the cure of all other diseases, or aggravated them. Preparations were therefore made to prevent and check it, and, by the judicious liberality of the captain, about 4000lbs. of vegetables, 2000lbs. of pickles made on-board, twelve dozen fine capons, and six bullocks, were purchased and carried to sea, for the use of the sick and scorbutics. On the 14th of February, the Belliqueux sailed from Macao, with a convoy of seven Indiamen, and were transferred by a fair wind in four days from a cold to a torrid climate, which made it agreeable to exchange the British woollens for the light calicoes of India and the nankeens of China. On the 2d of March the fleet arrived in the straits of Banca, and took in water and wood

* Scorbutus. "Invenitur apud Britannos, Batavos, Suecos, Danos, Norvegos, Germanos, &c. attingit adeaque *Boreales populos et frigidiori sub climate viventes.*—*Boerhaave, Aphor.* 1150.

"In regione frigida, post victum putrescentem, salitum," &c.—*Nosologia Culleni. Classis III. Ordo III. Scorbutus.*

at at the Nanka Islands, a service in which ten men were sunburnt, i. e. blistered by the heat of the sun on the back, face, and arms: these vesications were extensive, and, as they precisely resemble the vesications of scalds from other causes, they were treated as such, and cicatrized with equal facility and rapidity. Cold applications and purgatives should be first employed, and, when the vesications have ruptured or are opened, the Cerat. Lapid. Calam. is the best dressing. On the 7th of March, the fleet reached the straits of Sunda, but were prevented by adverse winds and currents from clearing them until the 16th. The weather in both straits was very hot and sultry, and Fahrenheit's thermometer ranged from 84° to 94° at mid-day.

The most prevalent complaints, during this period, were such as arose from an increased secretion of the hepatic system, producing bilious fever, bilious redundance, and diarrhœa; a few cases of dysentery and phlegmon were met with.

After clearing the Straits of Sunda, the course of the fleet was directed across the South Pacific Ocean, and we experienced fine weather and a pleasant passage, until we reached the "stormy* spirit of the Cape," where the fleet encountered successive gales of wind, that dispersed all the convoy: the men endured many hardships, and sustained much labor and fatigue, before his Majesty's ship doubled the Cape of Good Hope in safety; for she had been four days in imminent danger of foundering, and was literally going to pieces, when a fair wind and current impelled us towards the Island of St. Helena, which we reached on the 25th of May. During our passage, between the Straits of Sunda and St. Helena, scurvy was the most prevalent disease, especially during the fatigues off the Cape; several cases of dysentery, increased secretion of bile, and complaints from cold, occurred. During the voyage from China to St. Helena, 133 men had been placed on the sick list, and on our arrival the number of scorbutics amounted to 60, notwithstanding several had been cured during the voyage.

The arrangements to prevent the access, and counteract the effect, of scurvy, were these: Every man on the sick list, or who had the slightest wound or ulceration, or who had manifested a predisposition to scurvy on former voyages, was supplied with one pound of potatoes or other vegetables, on four days in the week, and an unlimited allowance of pickles with his salted meats, on the other three days. The pomkins, with lime-juice, orange peel, and spices, make good

* Pleasures of Hope.

mockmock-apple pies. When a bullock was killed, or a donation of a quarter of mutton came from the captain, all above described were supplied with fresh meat and soup, and abundance of vegetables. The capons, with onions, pomkin, and rusk, made excellent soup, and a light diet for the worst cases of disease.

Agreeable to the naval regulations, every man was served with punch daily, or wine and sherbet. Four ounces of lime-juice were prescribed daily, and sometimes eight, to those much afflicted with scurvy. The greatest attention to the early use of these prophylactics, did not prevent several cases of scurvy from becoming worse, during the voyage, when the supply of fresh animal food was scanty, but these soon recovered at St. Helena; thus demonstrating clearly, on this as well as many former occasions, that there is no certain cure for, or preventative of, scurvy, but a *full diet of fresh animal and vegetable food.* The disease was, however, prevented from assuming its most fatal forms, and was much arrested in its progress.

At St. Helena, the supplies of vegetables are good, abundant, and dear, but it is well known that the live stock is under the protection of the governor in council, and is only killed by their order. From earnest representations and much address, the Belliqueux obtained more bullocks and sheep than had been ever given to one ship, and the government supply of vegetables was liberal and ample. In the clear rivulet that meanders through James's Valley, the water-cress abounds, and the convalescents and scorbutics derived much benefit from their rambles along its banks, and eating the vegetable from its pure source: they also gathered large quantities for their shipmates on board.

The health and strength of the people were much recruited, and the scorbutic disposition corrected, by the salutary refreshments obtained at the island, from whence the fleet departed on the 9th of June, with the addition of two frigates and five sail of Indiamen.

Nearly as large a proportion of vegetables and pickles were appropriated to the prevention and cure of diseases, as had been set apart for sea use in China, and similar arrangements in their distribution were adopted. The enormous price of ten shillings for a fowl, together with their scarcity, precluded the possibility of purchasing so large a stock of poultry as was obtained in China; some Cape sheep were obtained to supply the deficiency.

The fleet touched at the Island of Ascension, where turtle enough were turned to give the ship's company turtle-soup two days: from thence the course was steered to the north-

5 ward ward and westward of the Western Islands, and, after passing them, the course was shaped for England, where the fleet arrived, and anchored in the Downs, on August 8th, 1811. During the passage from St. Helena to England, ninety-one patients were placed on the sick list, and some cases of scurvy were met with: the most prevalent diseases were dysentery, bilious fever, and (in the latter part of the voyage) scurvy. It affords no small gratification to observe, that not a single case of disease terminated fatally, during this long voyage of six months, performed in every variety of climate and diversity of temperature, which prevail between 50° of north, and 40° of south, latitude. The only death on board occurred from accident; a marine boy in a state of intoxication fell down the hatchway, fractured one of the cervical vertebræ, and died the next day. The successful preservation of the lives of the seamen, during this voyage, must be principally ascribed to the means adopted for preventing the progress of scurvy; for, when a sailor is affected with this disease, he is literally in famine in the midst of plenty. He avoids his salted meats as a known poison, which slowly corrupts his blood, and destroys his vital powers; while the digestive functions become so much weakened, as to be incapable of assimilating sufficient nutrition from the pease, oatmeal, bread, wine, and lime-juice, which constitute the other principal ingredients of his fare, as fatal experience has too often proved.

It is singular, that a wise government of a commercial nation does not establish some distinction between the diet of a sick and a healthy sailor, and furnish by regulation, for the convalescent and scorbutic, those refreshments for which the seaman now depends upon the precarious liberality and humanity of his officers; for the necessity of a full diet of fresh animal and vegetable food in the cure of scurvy cannot be too strongly urged, and every ship should be liberally provided with it on all long voyages.

*H. M. S. Warrior*, R. W. BAMPFIELD.

*off Flushing*, *August* 10, 1812.

